# Neuroprotective Effects of Isosteviol Sodium in Murine Brain Capillary Cerebellar Endothelial Cells (cerebEND) After Hypoxia

**DOI:** 10.3389/fncel.2020.573950

**Published:** 2020-10-28

**Authors:** Nils Rösing, Ellaine Salvador, Paul Güntzel, Christoph Kempe, Malgorzata Burek, Ulrike Holzgrabe, Vladimir Soukhoroukov, Christian Wunder, Carola Förster

**Affiliations:** ^1^Department of Anesthesia and Critical Care, Division Molecular Medicine, University of Würzburg, Würzburg, Germany; ^2^Tumor Biology Laboratory, Department of Neurosurgery, University of Würzburg, Würzburg, Germany; ^3^Institute of Pharmacy and Food Chemistry, Biocenter, University of Würzburg, Würzburg, Germany; ^4^Department of Biotechnology and Biophysics, Biocenter, University of Würzburg, Würzburg, Germany; ^5^Department of Anesthesia and Intensive Care Medicine, Robert-Bosch Hospital, Stuttgart, Germany

**Keywords:** isosteviol sodium, hypoxia, cerebEND cells, blood brain barrier, neuroprotection

## Abstract

Ischemic stroke is one of the leading causes of death worldwide. It damages neurons and other supporting cellular elements in the brain. However, the impairment is not only confined to the region of assault but the surrounding area as well. Besides, it also brings about damage to the blood-brain barrier (BBB) which in turn leads to microvascular failure and edema. Hence, this necessitates an on-going, continuous search for intervention strategies and effective treatment. Of late, the natural sweetener stevioside proved to exhibit neuroprotective effects and therapeutic benefits against cerebral ischemia-induced injury. Its injectable formulation, isosteviol sodium (STVNA) also demonstrated favorable results. Nonetheless, its effects on the BBB have not yet been investigated to date. As such, this present study was designed to assess the effects of STVNA in our *in vitro* stroke model of the BBB.The integrity and permeability of the BBB are governed and maintained by tight junction proteins (TJPs) such as claudin-5 and occludin. Our data show increased claudin-5 and occludin expression in oxygen and glucose (OGD)-deprived murine brain capillary cerebellar endothelial cells (cerebEND) after STVNa treatment. Likewise, the upregulation of the transmembrane protein integrin-αv was also observed. Finally, cell volume was reduced with the simultaneous administration of STVNA and OGD in cerebEND cells. In neuropathologies such as stroke, the failure of cell volume control is a major feature leading to loss of cells in the penumbra as well as adverse outcomes. Our initial findings, therefore, point to the neuroprotective effects of STVNA at the BBB *in vitro*, which warrant further investigation for a possible future clinical intervention.

## Introduction

As one of the leading causes of death worldwide, stroke has become a global medical problem that requires an on-going, continuous search for intervention strategies and effective treatment (Moskowitz et al., 2010). Between the two different types of stroke, ischemic, and hemorrhagic, the former accounts for the majority of all strokes (Silvestrelli et al., 2002; Rammal and Almekhlafi, 2016). Ischemic stroke results from insufficient cerebral blood flow caused by occlusion or stenosis of a brain artery (Ovbiagele et al., 2013). Due to the assault to the brain during a stroke, the blood-brain barrier (BBB) is damaged and compromised leading to edema, microvascular failure, and subsequent neuronal cell death (Shi et al., 2019).

Cerebral edema is increased water accumulation in brain tissue, including individual cells as well as surrounding interstitial space (Winkler et al., 2016). It is classified as either vasogenic or cytotoxic, although they appear to be interrelated. Vasogenic edema occurs when extracellular fluid resulting from BBB disruption accumulates and serum proteins are extravasated. Meanwhile, cytotoxic edema emerges from cell swelling caused by the build-up of intracellular fluid (Michinaga and Koyama, 2015).

Within the ischemic cerebrovascular bed, there are two major zones of injury: the core ischemic zone and the “ischemic penumbra” (ischemic but still viable cerebral tissue). In the core zone, which is an area of severe ischemia, the loss of oxygen and glucose (OGD) results in the rapid depletion of energy stores. Severe ischemia can result in necrosis of neurons, supporting cellular elements (glial cells) and cells of the BBB within the severely ischemic area. Brain cells within the penumbra, a rim of mild to moderately ischemic tissue lying between tissue that is normally perfused and the area in which infarction is evolving, may remain viable for several hours (Thirugnanachandran et al., 2018). That is because the penumbral zone is supplied with blood by collateral arteries anastomosing with branches of the occluded vascular tree. However, even cells in this region will die, due to the following phenomenon. First, cerebral edema occurs and reperfusion is not established during the early hours since the diffusion length for oxygen remains too long and collateral circulation is inadequate to maintain the permanent neuronal demand for OGD indefinitely (Heiss, 2012; Wu et al., 2018). Second, the so-called ischemia-reperfusion injury. Reperfusion (recovery of oxygen supply in former ischemic tissue) leads to harmful reactive oxygen species (ROS) production and oxidative stress, which is responsible for most of the ischemia-reperfusion injury and thus causing brain tissue damage (e.g., apoptosis, autophagy, and necrosis) (Rodriguez et al., 2020). Since brain edema is fatal, but only symptomatic treatments to eliminate edema fluid are currently at hand, the development of novel therapeutic strategies is necessary.

Of the many currently available natural products used for many years as traditional medicine, stevioside, which is a natural sweetener extracted from *Stevia rebaudiana* Bertonileaf is worth the attention (Ghisalberti, 1997; Kinghorn and Soejarto, 2002; Bassoli and Merlini, 2003). Its injectable formulation, isosteviol sodium (STVNA) salt, has shown potential as an emergency treatment due to its high solubility and bioavailability (Lai et al., 2017). Moreover, it has recently been demonstrated to exhibit neuroprotective effects and therapeutic benefits against cerebral ischemia-induced injury (Hui et al., 2016; Zhang et al., 2017, 2018). Nonetheless, its effects on the BBB have not yet been investigated to date.

Because tight junction proteins seal the spaces between capillary endothelial cells, they account for the integrity of the BBB (for reviews, see Salvador et al., 2014, 2016). Compromised BBB integrity equals increased permeability. More often than not, this involves changes in TJ protein expression such as claudin-5 and occludin (Luissint et al., 2012; Stamatovic et al., 2016). Meanwhile, integrins, heterodimeric transmembrane proteins with α, and β sub-units are also reported to play a crucial role in BBB integrity. It is demonstrated that modulation of integrins as matrix adhesion receptors can lead to barrier changes during brain injuries (del Zoppo and Milner, 2006).

Considering that the series of events that take place following ischemia are majorly due to loss of glucose and oxygen, oxygen-glucose deprivation (OGD) is the method of choice in modeling stroke *in vitro* (Salvador and Förster, 2014). Meanwhile, our murine brain microvascular endothelial cells (cerebEND) derived from the cerebellum is an established model of the blood-BBB (Silwedel and Förster, 2006). Therefore, subjecting these cells to OGD provides us with an ideal model of stroke and at the same time allows us to study its effects involving the BBB. Hence, in this study, we investigate the neuroprotective effects of STVNA on the BBB during stroke using our established *in vitro* model system.

## Materials and Methods

### Cell Culture

Murine microvascular brain endothelial cells (cerebEND) (Silwedel and Förster, 2006) grown in Dulbecco’s Modified Eagle’s Medium (DMEM) supplied with 50 U/ml penicillin/streptomycin and 10% fetal calf serum (FCS) were seeded in six-wells plate (Greiner Bio-One GmbH) with a density of 40,000 cells/ cm^2^ and cultured in a 37°C incubator (Steri-Cult 200, Forma Scientific) until 95% confluent as observed under the microscope. Each experiment consisted of triplicate wells for every treatment and was repeated at least thrice.

### Oxygen Glucose Deprivation (OGD) and Isosteviol Sodium (STVNA) Treatment

Upon confluence, the cells were differentiated in DMEM medium with 1% FCS for 24 h. Afterwards, the medium of the OGD treatment group was changed into glucose and pyruvate-free DMEM before placing them for 4 h in the OGD chamber (Heracell 150i, Thermo Fisher Scientific, Waltham, MA, USA) with the following conditions: 37°C, 5% CO_2_, and 1% O_2_ (Kleinschnitz et al., 2011; Salvador et al., 2018). Next, cells were treated with 0, 1, 5, 10, and 20 mg/l STVNA for 4 and 24 h in a 37°C incubator post OGD. The various concentrations of STVNA were obtained by weight (salt) per volume (medium) dilutions.

### Isosteviol Sodium Synthesis

Chemicals were obtained from Carboluten Chemicals GmbH (St. Ingbert, Germany) and Sigma–Aldrich (Schnelldorf, Germany) and were used without further processing. H (400.132 MHz) NMR spectra were recorded on a Buker AV400 instrument (Bruker Biopsin). The signal of a deuterated solvent was used as an internal standard (DMSO-d6: 2.5 ppm). Isosteviol was dissolved in 70 ml tetrahydrofuran (THF) in which 8 M NaOH was later added. The reaction mixture was stirred at room temperature for 30 min. After the solvent was removed under reduced pressure, the isosteviol sodium was obtained as a white salt (680 mg, 2.0 mmol, 95%).

### Quantitative Real-Time Polymerase Chain Reaction (qRT-PCR)

Total RNA was isolated from lysed cells and purified according to the manufacturer’s instructions (Nucleospin RNAII, Machery- Nagel). Next, the generation of cDNA was carried out using the High Capacity cDNA Reverse Transcriptase Kit (Thermo Fisher Scientific, Waltham, MA, USA). One microgram cDNA was used for qRT-PCR with Taq Man Fast Advanced Master Mix (Applied Biosystems). The following TaqMan Gene Expression Assay primers were used: claudin 5 (Mm00727012_s1), integrin α1 (Mm01306375_m1), integrin αv (Mm00434486_m1) and Canx (Mm00500330_m1) (Applied Biosystems). The 2^−ΔΔCT^ method was used for quantification. Meanwhile, fold expression was used for graphic representation with the endogenous control Canx set as reference.

### Western Blot

Cells were washed twice with cold phosphate-buffered saline (PBS) and lysed with RIPA buffer (50 mM Tris pH 8.0, 150 mM NaCl, 0.1% SDS, 0.5% sodium deoxycholate, 1% NP40) containing protease inhibitor cocktail (Roche) and Phenylmethylsulfonylfluoride (PMSF). Next, cells were sonicated (Bandelin SONOPULS) and mixed with Laemmli buffer containing 5% β-mercaptoethanol. After denaturation, they were run through a 12% SDS PAGE mini gel and blotted overnight using a Mini Trans-Blot Electrophoretic Transfer Cell (BioRad). Subsequently, the membrane was blocked in 5% non-fat dry milk (Carl Roth) and probed with the primary claudin-5 (1:500, Invitrogen), occludin (1:100, Acris), and β-actin (1: 25,000, Sigma–Aldrich), followed by secondary antibodies anti-mouse/rabbit (1:3,000, Roche Lumi Light Plus and Cell Signaling Technology) and anti-guinea pig (1:5,000, Santa Cruz). Detection was carried out using an electrochemiluminescence solution (Whitehead et al., 1979) and viewed with Imagen Flour Chem FC2 (Cell Biosciences) with the AlphaView Software (Version 1.3.0.7, Innovatech Corporation). Densitometric analyses were performed using ImageJ software (NIH and LOCI, University of Wisconsin).

### Optical Volume Measurement

To analyze the effects of isosteviol sodium (STVNA) on the volume of cerebEND cells following OGD/normoxia, the optical volume was measured (Andronic et al., 2015). Briefly, cells were simultaneously subjected to OGD and treated with 10 and 20 mg/l STVNA. Afterwards, the optical volume measurement was carried out as quickly as possible. Briefly, cells were washed twice with PBS (Sigma–Aldrich, Schnelldorf, Germany) and detached with 300 μl of trypsin (Biochrom). 1.5 ml of cell culture medium were then added per well and the respective cell suspension was transferred into a 50 ml Falcon tube. Afterward, 10 ml of the suspension was placed into a volumetric chamber using a syringe. The cells were visualized on a screen with a CCD camera (uEYE, IDS GmbH, Obersulm, Germany) attached to a light microscope (Leica Leitz DMRM, Hamburg, Germany) *via* the uEye software (IDS GmbH, Obersulm, Germany). Images captured were quantified using ImageJ (NIH and LOCI, University of Wisconsin).

### Statistical Analysis

Data were analyzed using *t*-test and ANOVA with *p* < 0.5 considered as significant. Statistical analyses were made using the GraphPad PRISM 7 software (GraphPad Software, Inc.).

## Results

### Alterations in Tight Junction Proteins Expression After Oxygen-Glucose Deprivation

Tight junction proteins (TJPs) influence the unique barrier properties of brain capillary endothelial cells making up the BBB. When the BBB is damaged and compromised, the expression of these TJPs could be altered such as what occurs during ischemia. Therefore, after subjecting the cerebEND cells to oxygen-glucose deprivation (OGD), they were treated with various concentrations (0, 1, 5, 10, and 20 mg/l) of isosteviol sodium (STVNA) for 4 and 24 h. Afterward, expression levels of claudin-5 and occludin were evaluated by western blot analyses.

**Figure 1:**
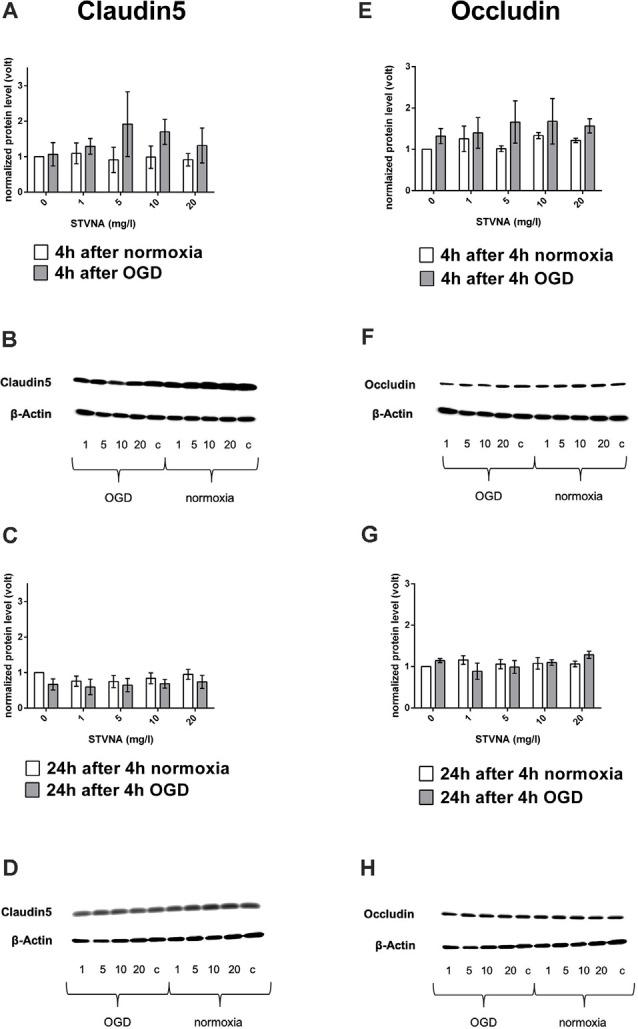
Western blot and densitometric analyses of claudin-5 **(A–D)** occludin **(E–G)** after oxygen-glucose deprivation (OGD; **A,E**) and subsequent (isosteviol sodium, STVNA) treatment for 4 h **(A,B,E,F)** and 24 h **(C,D,G–H)**. Representative Western blot depicting claudin-5 **(B,D)** and occludin **(F,H)** expression. Values shown are the means of three to five independent experiments normalized to β-actin. Statistical significance was evaluated using unpaired *t*-test, Tukey’s multiple comparison test, *c* = control.

Results show increased claudin-5 expression after treating the cells with STV Na for 4 h after 4 h OGD with 5 mg/l yielding the highest increase (Figures 1A,B). On the other hand, claudin-5 expression decreased after STVNA treatment for 24 h after 4 h OGD (Figures 1C,D). Similar findings were observed for occludin (Figures 1G,H). However, the highest increase in expression after 4 h STVNA treatment following 4 h OGD was with both 5 and 10 mg/l concentrations (Figures 1 E,F).

**Figure 2:**
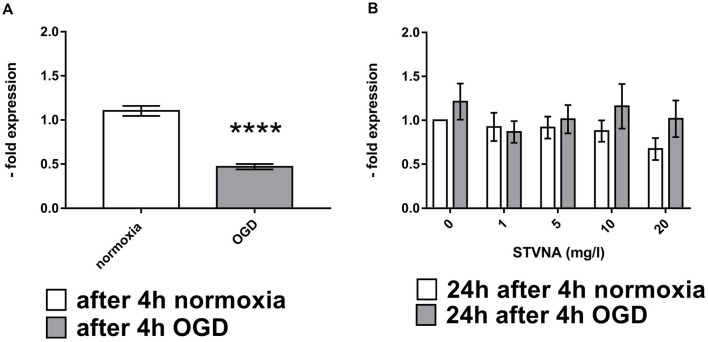
Quantitative Real-Time Polymerase Chain Reaction (qRT-PCR)analysis of claudin-5 mRNA expression after OGD **(A)** and subsequent STVNA treatment for 24 h **(B)**. Values shown are the means of 4 independent experiments normalized to Canx. Statistical significance was evaluated using unpaired *t*-test, Tukey’s multiple comparison test, ****p < 0.0001.

Similarly, expression of claudin-5 was also analyzed at the mRNA level through qRT-PCR, following 4 h OGD and subsequent STVNA treatment for 24 h. Subsequent treatment with STVNA for 24 h post-OGD resulted in increased claudin-5 expression for all STVNA concentrations tested (Figure 2).

### OGD and STVNA Influence on the Transmembrane Protein Integrin Expression

Like TJPs, integrins are a central component of the cell to cell contacts. Besides, however, they also form cell and matrix associations. To test the influence of STVNA on inflammatory parameters, the expression of integrin_α1_ and integrin_αv_ after 4 h OGD followed by treatment with 0, 1, 5, 10, and 20 mg /l STVNA for 4 and 24 h has been analyzed using qRT- PCR.

**Figure 3:**
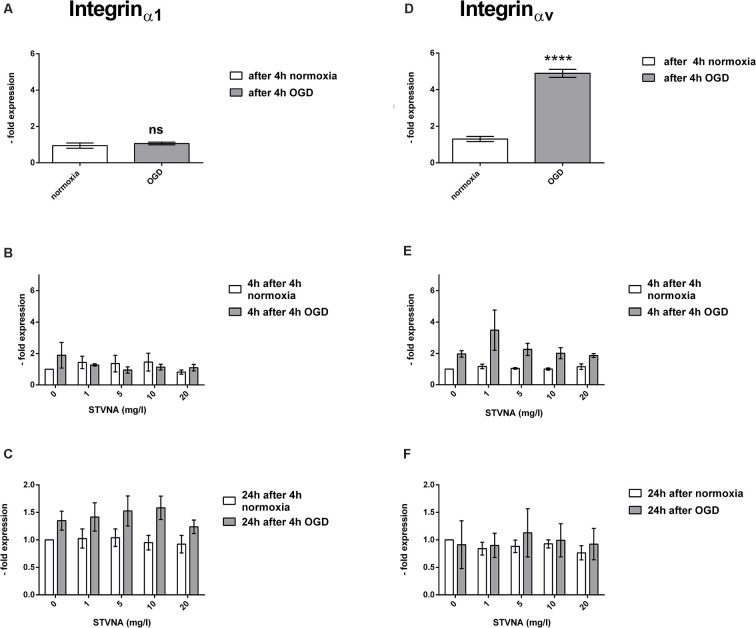
qRT-PCR analysis of integrin_α1_ **(A–C)** and integrin_αv_ **(D–F)** mRNA expression after OGD **(A,D)** and subsequent STVNA treatment for 4 h **(B,E)** and 24 h **(C,F)**. Values shown are the means of four independent experiments normalized to Canx. Statistical significance was evaluated using unpaired *t*-test, Tukey’s multiple comparison test, ****p < 0.0001. ns = not significant.

A significant increase in the mRNA expression of integrin_αv_ was observed in OGD-treated cerebEND cells (*p* < 0.0001). On the other hand, no significant change was demonstrated by integrin_α1_. Likewise, although no significant difference was shown by the qRT-PCR data on the effects of STVNA treatment for both 4 and 24 h incubation time points, there is a marked increase in integrin_αv_ expression after 4 h of STVNA treatment at a concentration of 1 mg/l, following OGD. Likewise, a similar trend of increased integrin_α1_ expression after 24 h of STVNA treatment after OGD in all STVNA concentrations administered could be observed (Figure 3).

### Effects of OGD and STVNa on the Optical Cell Volume

**Figure 4:**
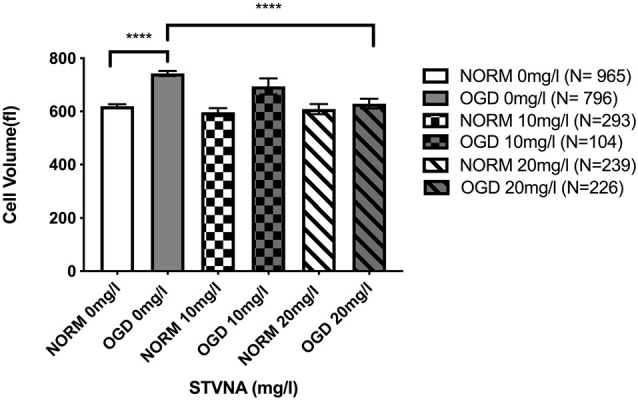
Optical volume measurement of cerebEND cells subjected to 4 h OGD coupled with simultaneous 10 and 20 mg/l STVNA treatment. *N* = number of cells included in the measurement with a total of 2,326 cells. Statistical significance was evaluated using unpaired *t*-test, Tukey’s multiple comparison test, ****p < 0.0001.

Using a flow chamber, the optical volume of cerebEND cells subjected to OGD coupled with simultaneous treatment using 10 and 20 mg/l STVNA was examined. THE untreated OGD group demonstrated a significant volume increase of 19.87% (*p* < 0.0001) compared to the untreated normoxia group. Meanwhile, upon treatment with 20 mg/l STVNA, a significant volume reduction of 15.28% (*p* < 0.0001) was observed in the treated OGD group compared to the untreated OGD group (Figure 4). In the same manner, volume reduction was observed in the OGD group treated with 10 mg/l STVNA compared to the untreated OGD group, although not statistically significant (*p* < 0.47). Meanwhile, no significant volume change was displayed by STVNA treated compared to untreated cerebEND cells under normoxic conditions.

## Discussion

Neuroprotection includes mechanisms and strategies aiming to protect the nervous system from injury and damage, especially in people who sustain an injury or develop a health condition that has neurological effects. Although previous reports demonstrated neuroprotective benefits of STVNA against cerebral ischemia-induced injury in rats (Hui et al., 2016; Zhang et al., 2018) and hypoxia in neuroblastoma cells (Zhong et al., 2019), to our knowledge, we are the first to study the effects of STVNA in an *in vitro* blood-BBB model of cerebral ischemia. Albeit our model comprises of but a single cell type, it is proven and established to suffice in mimicking ischemic conditions (Neuhaus et al., 2012, 2014). Our preliminary findings that STVNA administration after or during oxygen-glucose deprivation (OGD) increases expression of tight junction proteins claudin-5 and occludin and reduces cell volume, respectively, point to neuroprotective benefits of STVNA to the BBB *in vitro*. Since, the integrity of the BBB plays a crucial role in the occurrence of cerebral edema (especially in the penumbra, where viable cells “await” OGD for sufficient energy metabolism), investigating whether the same effects can be observed therein could prove profitable in designing future strategies to alleviate such devastating problems faced by stroke patients. For this reason, our study is a preliminary attempt to explore how STVNA affects the BBB concerning ischemia.

During cerebral ischemia, endothelial cells of the BBB are vitally threatened. There is not only structural loss within endothelial cells but a predominance of endothelial edema in the vessels also takes place (Krueger et al., 2015, 2017). This eventually leads to long-lasting vascular damage. BBB breakdown results in life-threatening complications and is a common cause of death. During ischemia, primarily occurring brain damage is unavoidable, which is why the treatment of secondary brain damage should be in the foreground. Since no optimal therapy for the treatment of secondary brain damage has been developed to date, approaches geared towards endothelial protection may help to reduce vasogenic edema after ischemic stroke.

In our study design, the treatment period of 0 h directly after 4 h OGD was used to demonstrate the direct effect of the OGD procedure without treatment with STVNA. The treatment periods of 4 h and 24 h, meanwhile, were employed to examine a possible effect of STVNA in the clinical acute phase after a cerebral ischemic event *in vitro*. The increased protein expression of both claudin-5 and occludin 4h-post STVNA treatment at 5–10 mg/l concentration, following 4h-OGD, is a possible indication of STVNA stabilizing effects within the first 4 h of STVNA therapy. After 24 h, the leveling out of the protein expression levels in the STVNA-treated group with that of the control group points to a 4-hour therapeutic window of STVNA. This is following the findings by Hui et al. (2016) where, favorable effects of STVNA were still present 4 h post-administration, after the recirculation in transient ischemia or 4 h after artery occlusion in permanent ischemia. The neurological damage after stroke can be permanent. Hence, prompt diagnosis and treatment are required for its prevention. In this regard, taking into account the therapeutic window of any drug is crucial in the generation of novel therapeutic strategies and drug design. For instance, the tissue plasminogen activator (tPA), which is currently the only therapeutic agent approved to treat patients with acute ischemic stroke, manifests clinical benefits within 4.5 h of stroke onset (Kim, 2019). Accordingly, concerns for hemorrhagic complications and the requires that tPA be administered within 3 h of symptoms. Beyond this, the integrity of the BBB is compromised (Su et al., 2008). Moreover, relevant animal studies suggest that irreversible focal injury begins within a few minutes and is complete within about 6 h (Zivin, 1998).

Similarly, the upregulation of integrin-αv mRNA expression 4 and 24 h after STVNA treatment of OGD-subjected cerebEND cells indicates increased BBB integrity due to STVNA treatment. Correspondingly, the decreased expression of integrin_α1_ is a confirmation of such. Neuroinflammation could lead to reduced expression of integrin_α1_, the α-subunit of the major collagen receptor integrin_α1β1_, on microvascular endothelium responsible for endothelial cell adhesion to the matrix ligand collagen type IV (Defilippi et al., 1991). In assessing the effects of dexamethasone in inducing the expression of metalloproteinase inhibitor TIMP-1 in the murine cerebral vascular endothelial cell line cEND, it was reported that the downregulation of integrin_α1_ expression is inversely proportional to the elevated levels of the integrin_αv_ upon TNFα administration (Förster et al., 2007). Also, integrin αvβ3 was identified as a novel modulator of endothelial barrier enhancement (Su et al., 2012).

Finally, the reduction in cell volume upon simultaneous administration of STVNA and OGD indicates neuroprotective benefits of both 10 and 20 mg/l STVNA to cerebEND cells. One occurrence frequently associated with pathological states such as cerebral ischemia is first the occurrences of an ischemic penumbra and afterward a deleterious swelling of cells and consecutive increase in intracranial pressure (Kimelberg, 2000). In neuropathologies such as stroke, the failure of cell volume control is a major feature leading to loss of cells in the penumbra, and adverse outcomes (Wilson and Mongin, 2018).

At large, even though this study is limited to the preliminary findings of the effects of STVNA in the BBB, it strengthens the claim of already published literature about the neuroprotective properties of STVNA *in vivo* (Hui et al., 2016; Zan et al., 2018, Zhang et al., 2018). Hence, it points out to the possibility of STVNA to enable new therapeutic approaches in both the clinical and preclinical care of cerebral ischemic events. Therefore, together with this, the initial findings in this study pointing to the neuroprotective effects of STVNA at the BBB *in vitro* warrant further investigation for a possible future clinical intervention. STVNA should further be explored as a potential drug therapy of cerebral ischemic events.

## Data Availability Statement

The raw data supporting the conclusions of this article will be made available by the authors, without undue reservation.

## Author Contributions

NR and ES performed and analyzed experiments and drafted the manuscript. PG and CK performed experiments. MB, UH, VS, CW, and CF supervised the project. CF and CW conceptualized and designed the study. All authors contributed to the article and approved the submitted version.

## Conflict of Interest

The authors declare that the research was conducted in the absence of any commercial or financial relationships that could be construed as a potential conflict of interest.
